# Diagnostic Potential of Anterior Segment Optical Coherence Tomography Scans for *Pseudomonas* Keratitis

**DOI:** 10.1167/tvst.12.11.34

**Published:** 2023-11-29

**Authors:** Haidar Khalil, Matthias Bolz, Klemens Waser, Leon Pomberger, Peter Laubichler, Paul Jirak, Nino Hirnschall

**Affiliations:** 1 Department of Ophthalmology and Optometry, Kepler University Clinic GmbH, Johannes Kepler University, Linz, Austria; 2 Medical Faculty, Johannes Kepler University, Linz, Austria

**Keywords:** keratitis, anterior segment OCT, *Pseudomonas* keratitis

## Abstract

**Purpose:**

The purpose of this study was to investigate the diagnostic value of anterior segment optical coherence tomography (AS-OCT) scans for *Pseudomonas* keratitis.

**Methods:**

Patients with treatment-naïve keratitis underwent AS-OCT imaging. The following parameters were evaluated: corneal thickness (CT), infiltrate thickness (IT), infiltrate diameter (ID), tissue loss/gain, entropy, and distance of the lesion from the corneal pupillary center. Three different OCT devices were used for the analysis. The relationship between the detected pathogen and the OCT patterns was analyzed.

**Results:**

Nineteen eyes of 19 patients were included in the analysis: seven cases in the *Pseudomonas* group and 12 cases in the Gram-positive group. The mean (SD) values for the *Pseudomonas* and Gram-positive groups, respectively, were as follows: CT, 834 µm (165 µm) and 760 µm (120 µm); IT, 290 µm (152 µm) and 287 µm (84 µm); ID, 2067 µm (1470 µm) and 1307 µm (745 µm); distance to center, 3.0 mm (1.2 mm) and 3.0 mm (1.6 mm); epithelial defect, 1193 µm (586 µm) and 484 µm (615 µm); tissue gain, +31% (19%) and +10% (12%); and entropy level, 4.0 (0.8) and 3.9 (1.1)

**Conclusions:**

This study introduces novel insights by identifying specific OCT parameters that distinguish *Pseudomonas* keratitis, including a 30% tissue gain. These findings align with earlier research that underscores the potential of OCT in differentiating various pathogens causing keratitis.

**Translational Relevance:**

The findings of this study could be used to develop new diagnostic strategies for *Pseudomonas* keratitis. The OCT findings could be used to develop new biomarkers for the infection.

## Introduction

Each year, worldwide, there are 2 million new cases of keratitis, a vision-threatening pathology. It is a medical and socioeconomic burden that has reached epidemic levels in several countries.^1^ In the United States, keratitis is responsible for 1 million clinical visits per year, at a cost of approximately $245 million.^2^ *Pseudomonas aeruginosa* is the most common Gram-negative bacteria causing a severe and vision-threatening keratitis.^3^^–^^5^ Untreated or delayed therapy leads to blindness and sometimes loss of the eye.

Standard of care for infectious keratitis treatment is the use of local broad-spectrum antibiotic therapy with known disadvantages, such as increasing resistance to antibiotics,^6^ changes in the ocular microbiome,^7^ and the risk of a gap for some bacteria. Development of a more specific and customized therapy would be beneficial, but there is a lack of fast, efficient, and inexpensive diagnostic testing to detect specific keratitis pathogens. At the moment, the diagnostic procedures being utilized have several disadvantages. Microbiological culture is usually available, but results are often not at hand for the first week after corneal scraping, and, additionally, the rate of negative culture results ranges between 34% and 62%.^8^ This delay in diagnosis, together with a low sensibility from microbiological cultures, has prompted further developments in such areas as confocal microscopy and polymerase chain reaction (PCR). Confocal microscopy has the disadvantage of low accessibility and high user dependency, and PCR is expensive and not always available.

Previous small case series showed that optical coherence tomography (OCT) imaging could be supportive in distinguishing among the different pathogens causing keratitis.^9^ Comparisons between Gram-negative and gram-positive bacteria revealed that Gram-negative infections lead to an increased corneal and infiltrate thickness compared to Gram-positive bacteria. The aim of this study was to investigate the use of anterior segment OCT (AS-OCT) scans to distinguish between *Pseudomonas* keratitis and Gram-positive bacterial keratitis by identifying specific OCT markers.

## Materials and Methods

This prospective study is part of the VICTORIA (Validation of Interleukins and other Cytokines Together with OCT Imaging for Rapid Infectious Keratitis Assessment) project. Patients were recruited from the outpatient clinic of the Department of Ophthalmology at the Kepler University Clinic, Linz, Austria. In our study, patients underwent screening within the initial 48 hours following symptom onset, thus ensuring early-stage imaging of the disease course. After clinical diagnosis of infectious keratitis, the patient was informed about the study and was included if written and oral consent was obtained. The study was approved by the local ethics committee (EK Nr: 1246/2021) and adheres to the tenets of the Declaration of Helsinki. Inclusion criteria were an untreated keratitis, written informed consent prior to study-related procedures, and age of at least 18 years at the time of inclusion.

### Measurements

Three different OCT devices were used to perform scans of the cornea: CASIA2 (Tomey, Tokyo, Japan), SPECTRALIS (Heidelberg Engineering, Heidelberg, Germany), and MS-39 (CSO, Firenze, Italy). Patients were placed in front of the devices and measurements were taken by trained staff. Also, slit-lamp photography of the infected eye was done. For the Heidelberg device, a separate mount was attached to scan the anterior segment of the eye. All scans were stored on site. After imaging with all three devices, corneal scraping was performed. Topical anesthesia (1% lidocaine) was placed in the affected eye, and the scraping was performed using a sterile needle or a sterile hockey knife. It was dissolved in a nutrient broth (without contamination of other surfaces) and sent to the microbiological laboratory.

### Image Analysis

**Figure 1.**
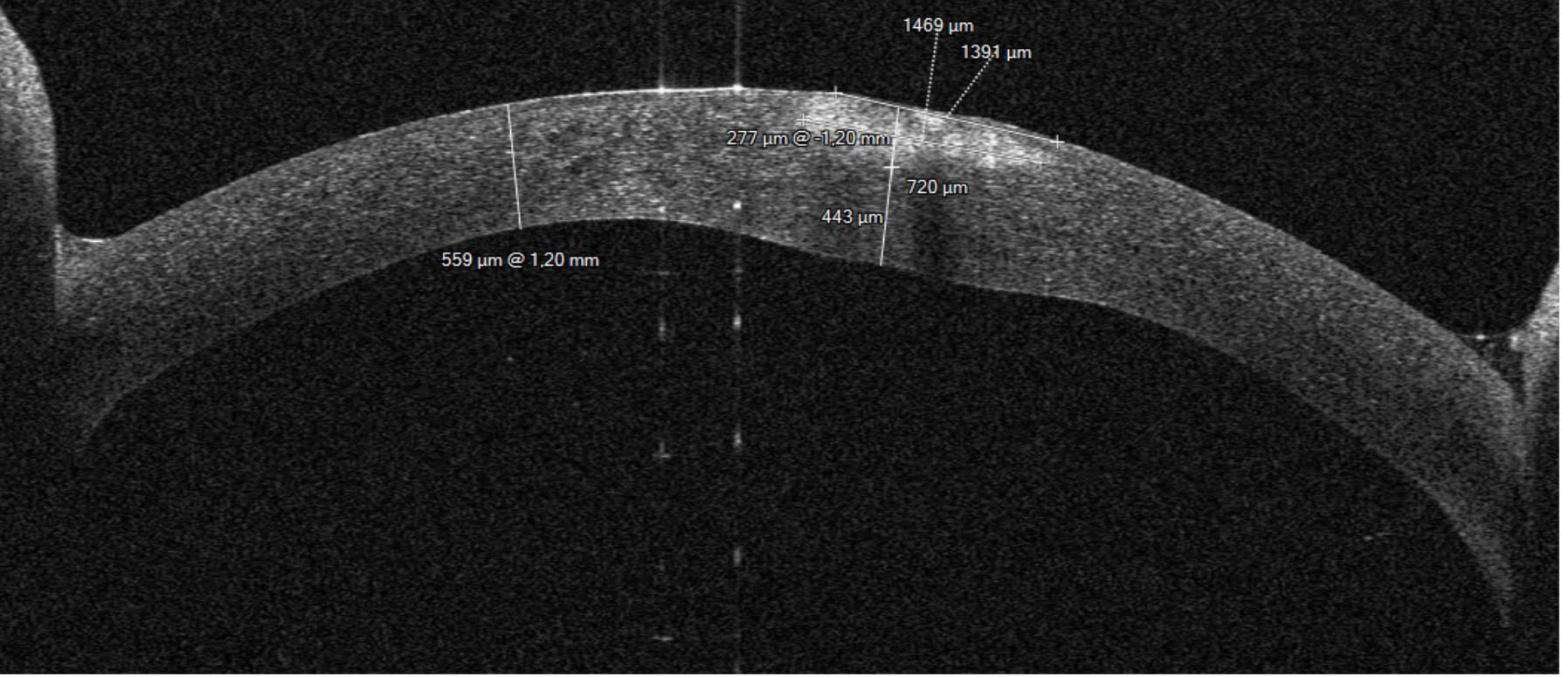
Tomey MS-39 AS-OCT values obtained using the built-in software.

**Figure 2.**
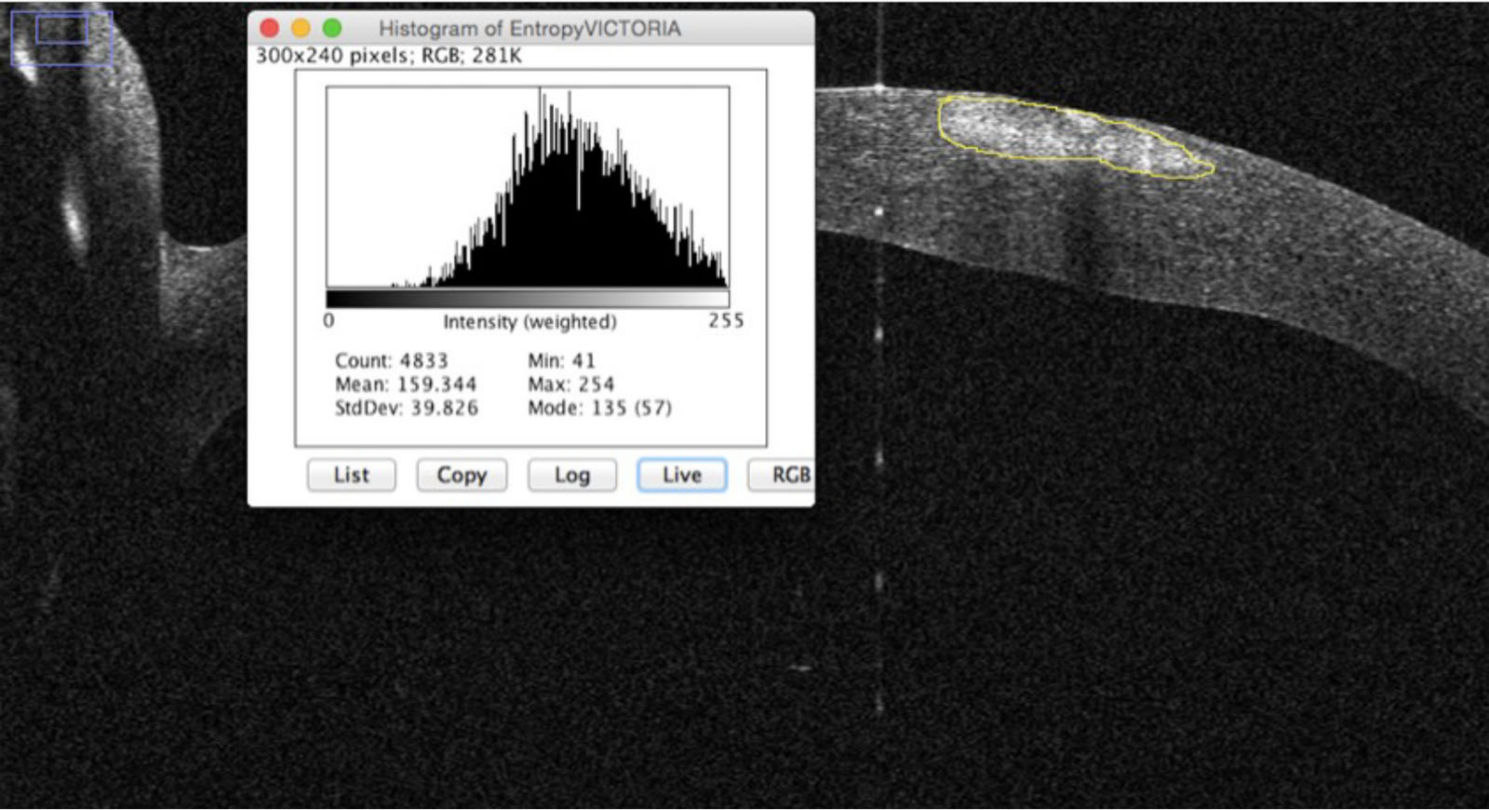
Grayscale histogram of the infiltrate area, marked with an ImageJ caliper.

**Figure 3.**
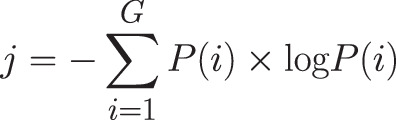
Formula used for calculating the entropy.

All images were analyzed using the device specific software and additionally ImageJ (National Institutes of Health, Bethesda, MD). The following parameters were analyzed using the respective built in programs of the devices: corneal thickness (µm), infiltrate thickness (µm), infiltrate diameter (µm), epithelial defect (µm), infiltrate distance to the corneal apex (mm), corneal thickness 180° opposite to the infiltrate (µm), ratio of tissue loss or tissue gain in the area of infiltrate compared to the healthy opposite side (same distance measured from corneal apex to healthy side of the cornea of the same eye), and effect on the pupillary axis (yes/no) (Fig. 1). For the analysis of entropy, the image was copied into ImageJ and the area of interest was marked with a caliper (Fig. 2). A histogram of the selected area was generated for further assessment. The data were copied into Excel (Microsoft, Redmond, WA), and the entropy was determined using the formula shown in Figure 3.

A skilled and masked reviewer (PL) with substantial clinical expertise in corneal diseases examined all slit-lamp photographs and their corresponding OCT images. The reviewer's task was to determine whether, based solely on these images, he could identify cases of *Pseudomonas* keratitis. A second masked reviewer (KW), also with notable clinical experience regarding corneal infectious diseases, examined all slit-lamp photographs and OCT images separately for comparison regarding the diagnostic rate between OCT and slit-lamp photography.

## Results

A total of 25 patients, each with a single infected eye, met the inclusion criteria of the study, and subsequent microbiology results confirmed their positive infections (as shown in Table 1). Patients included in our study were screened within the first 2 days after the onset of symptoms. Fourteen of the patients were male, and 11 were female. Mean age was 45.9 years (SD, 20.1; range, 20 (min) to 89 (max)). Out of those 25 eyes, six eyes had to be excluded (three cases had herpes simplex virus, one case had *Aspergillus fumigatus*, one case had *Serratia marescens*, and one case had a positive microbiological result from the contact lens but no positive result from the cornea). The remaining 19 eyes of 19 patients were included in the analysis: seven eyes in the *Pseudomonas* group and 12 eyes in the Gram-positive group (Table 2). Among the eyes in the *Pseudomonas* keratitis group, five of the seven infections were associated with contact lens use, whereas in the Gram-positive group, four of the 12 infections were associated with contact lens use.

**Table 1.** Features of all Eyes With Bacterial Keratitis

Pathogen	Number of Patients	Status
*Pseudomonas*	7	Included
Gram-positive	12	Included
Other	6	Excluded

**Table 2.** Pathogen Characterization of all Included Bacterial Keratitis Cases

Pathogen	Number of Patients	Group
*Pseudomonas* aeruginosa	7	*Pseudomonas*
*Staphylococcus epidermidis*	6	Gram-positive
Coagulase-negative staphylococci	4	Gram-positive
*Corynebacterium macginleyi*	1	Gram-positive
*Staphylococcus aureus*	1	Gram-positive

In all cases with *Pseudomonas* keratitis, a further analysis was performed: infiltrate diameters associated with contact lens use (mean ± SD, 754.6 ± 114.6 µm) and not associated with contact lens use (mean ± SD, 1005.0 ± 24.8 µm) were assessed and compared, and the differences were found to be significant (*P* = 0.039). Therefore, a partial least squares regression was developed that included the parameters epithelial defect, tissue gain, contact lens, corneal thickness, infiltrate diameter, infiltrate thickness, entropy, and distance to center. Contact lens use was found to be the third most relevant prediction parameter in this model (see Appendix).

### OCT and Image Analysis

**Figure 4.**
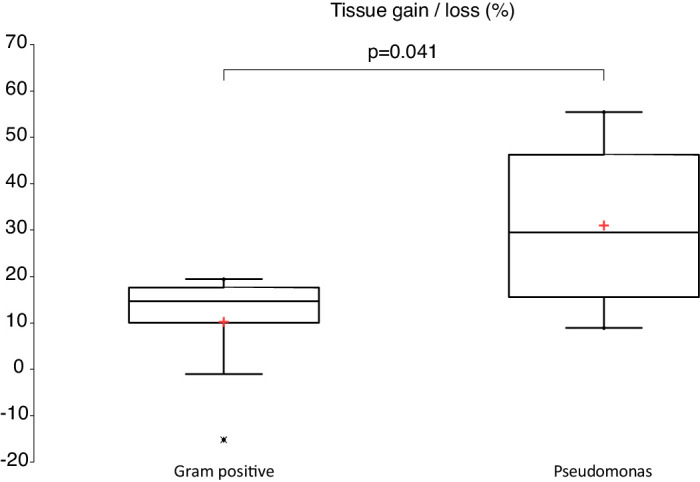
Box plot and whisker diagram showing a significantly higher tissue gain in the *Pseudomonas* group compared to the Gram-positive group (*P* = 0.041).

Median (mean ± SD) corneal thickness, infiltrate thickness, infiltrate diameter, and epithelial defect size for all patients were 782 µm (787 ± 139), 260 µm (288 ± 110), 1180 µm (1587 ± 1096), and 713 µm (868 ± 761), respectively. Median (mean ± SD) distance to the pupillary center, tissue loss/gain, and entropy were 2.95 mm (2.97 ± 1.38), 1.18% (1.22 ± 0.20), and 3.95 (3.92 ± 0.95), respectively. OCT findings comparing the *Pseudomonas* and the Gram-positive groups are summarized in Table 3. Tissue gain was found to be significantly higher in the *Pseudomonas* group compared to the Gram-positive group (30% vs. 15%; *P* = 0.041) (Fig 4).

In terms of the ratio of epithelial defect to infiltrate diameter, our analysis indicated a trend toward larger ratios in *Pseudomonas* cases (0.88 ± 0.56) in contrast to Gram-positive cases (0.49 ± 0.57), although this disparity did not reach statistical significance (*P* = 0.929).

Based on both OCT imaging and slit-lamp photography, masked reviewer one (PL) accurately recognized four of the seven instances of *Pseudomonas* keratitis. However, in three instances, cases of Gram-positive bacterial keratitis were misinterpreted as *Pseudomonas* keratitis. The other masked reviewer (KW) evaluated OCT and slit-lamp images separately. Three out of seven OCT images and four out of seven slit-lamp photographs were identified correctly as *Pseudomonas* keratitis. Five OCT images and four slit-lamp photographs of Gram-positive keratitis were misinterpreted as *Pseudomonas* keratitis.

**Table 3.** OCT Measurements of the *Pseudomonas* and Gram-Positive Groups

	Group, Median (Mean ± SD)		
OCT Finding	*Pseudomonas*	Gram-Positive	*P* (Mann–Whitney *U* Test)
Corneal thickness (µm)	853 (834 ± 165)	762 (760 ± 120)	0.374
Infiltrate thickness (µm)	229 (290 ± 152)	285 (287 ± 84)	0.967
Infiltrate diameter (µm)	1303 (2067 ± 1470)	1069 (1307 ± 745)	0.196
Distance to pupillary center (mm)	3.2 (3.0 ± 1.2)	3.0 (3.0 ± 1.6)	0.955
Epithelial defect (µm)	1051 (1193 ± 586)	252 (484 ± 615)	0.071
**Tissue gain (%)**	**+30 (31 ± 19)**	**+15 (10 ± 12)**	**0.041**
Entropy	4.1 (4.0 ± 0.8)	3.9 (3.9 ± 1.1)	0.958

Tissue gain values presented indicate a statistically significant difference between the Pseudomonas group (30%) and the Gram-positive group (15%).

### Prediction Model for *Pseudomonas* Detection

**Figure 5.**
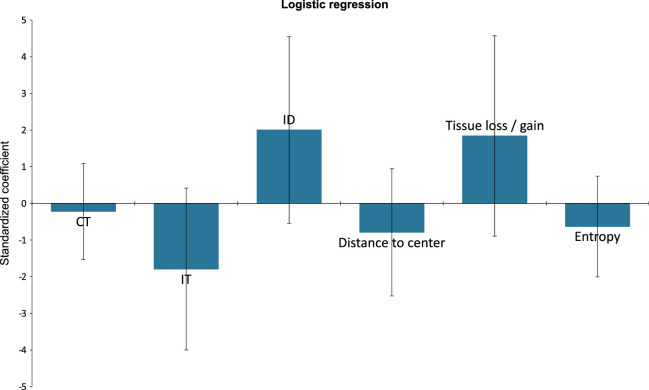
Logistic regression model showed significant impact of the following parameters for detecting *Pseudomonas* keratitis: infiltrate thickness (IT), infiltrate diameter (ID), and tissue loss/gain. Corneal thickness (CT), distance to center, and entropy were not statistically significant.

**Figure 6.**
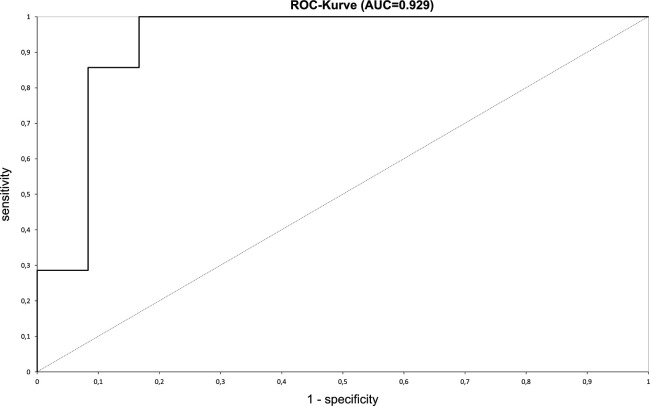
ROC curve for evaluating the sensitivity and specificity of following parameters: infiltrate thickness, infiltrate diameter, and tissue loss/gain. Area under the curve = 0.929.

Logistic regression was used to evaluate the influence of OCT findings on *Pseudomonas* detection. Infiltrate thickness (*P* = 0.009), infiltrate diameter (*P* = 0.026), and tissue loss/gain (*P* = 0.017) were found to be significant, whereas corneal thickness (*P* = 0.740), distance to center (*P* = 0.240), and entropy (*P* = 0.317) were not (Fig. 5). In the next step, only infiltrate thickness, infiltrate diameter, and tissue loss/gain were included in a logistic regression formula. The receiver operating characteristic (ROC) curve is shown in Figure 6. This model resulted in a relatively high sensitivity (85.7%) and a high specificity (91.7%). The area under the curve was 0.929.

## Discussion

This study shows the potential diagnostic value of AS-OCT scans in patients with *Pseudomonas* keratitis. The observed OCT patterns in the *Pseudomonas* keratitis group differed significantly from those of the Gram-positive group, even in this relatively small sample. A large infiltrate diameter and a gain of 30% of the corneal tissue appeared to be a predictive indicator for detecting *Pseudomonas* keratitis. A lower gain of 15% was observed in cases with Gram-positive bacteria. These results are in line with those of previous research, confirming that Gram-negative bacteria display a larger surface area of infiltration.^9^^–^^11^ A possible explanation for this may be the more severe inflammatory response of Gram-negative infections due to certain bacterial virulence factors.^12^^,^^13^ This hypothesis was also confirmed by Konstantopoulos et al.,^9^ who showed that, in Gram-negative infections, the tear film contains a higher quantity of interleukins than in Gram-positive infections. The Steroids for Corneal Ulcers Trial (SCUT) study analyzed the infiltrate and scar size of two different exotoxin-producing *Pseudomonas* strains.^14^ In our study, the parameter infiltrate diameter, which is most comparable to infiltrate and scar size, was much lower than in the SCUT trial. The mean diameter from our *Pseudomonas* keratitis group was 2067 µm compared to 4066 µm and 3061 µm (two strains) in the SCUT trial. This difference could be due to the fact that most patients in our trial were seen at an early stage of disease, whereas patients in the SCUT group may have been enrolled after several days or weeks of untreated keratitis. For the SCUT trial, the cornea was also measured with a slit lamp, whereas OCT provides a much more accurate measurement of infiltrate thickness because the infiltrate edges can be identified accurately.

Our study focused on early-stage *Pseudomonas* keratitis cases. Our patients were screened at an early phase of the infection, so the destructive enzymatic activity may not have yet fully manifested or caused extensive tissue damage. At this early stage, the infection might predominantly affect the corneal epithelium and anterior stroma, leading to localized changes that could be captured by AS-OCT imaging. As a result, a tissue gain parameter could potentially reflect the initial inflammatory response and tissue edema, rather than the later-stage destructive changes. Additionally, the tissue gain parameter we describe is relative to the corneal thickness of the opposite corneal side. This accounts for the potential variability in corneal thickness among individuals and provides a standardized metric for assessing changes within the context of the individual's own corneal thickness.

The variability in clinical features within *Pseudomonas* keratitis due to different strains is a crucial consideration when studying the complexity of this infection. Research has illuminated differences in presentations between cytotoxic and invasive strains of *Pseudomonas*, with notable distinctions observed across patient categories such as contact lens wearers and non–contact lens wearers.^15^ This variability extends to various aspects of the disease, including the size of corneal infiltrates. Notably, cytotoxic strains in contact lens wearers often manifest with smaller infiltrates compared to their invasive counterparts. The influence of contact lens use was also confirmed in this study. However, it is worth noting that our study did not specifically differentiate between *Pseudomonas* strains when investigating clinical parameters. The interplay between strain-specific pathogenicity and clinical presentations requires further exploration.

Corneal thickness, infiltrate thickness, infiltrate diameter, epithelial defect, and distance to center were not significantly different between *Pseudomonas* and Gram-positive keratitis. Except for the distance to the center, all of these parameters were raised, with a greater mean value leaning toward *Pseudomonas* but not reaching significance. A bigger sample size will be required to determine whether these parameters are beneficial for detecting *Pseudomonas* keratitis. Although some of those characteristics were proven to be effective for keratitis identification and progression in other investigations, clear guidelines for detecting the pathogen are lacking in the literature.^9^^,^^16^

The observed trend toward a larger ratio of epithelial defect to infiltrate diameter in *Pseudomonas* cases compared to Gram-positive cases suggests that there may be differences in the clinical presentation of these two types of infections. It also suggests that *Pseudomonas* infections might be associated with a relatively greater extent of epithelial damage in relation to the overall infiltrate size. This finding is in line with the commonly known nature of *Pseudomonas* keratitis, characterized by rapid progression and destructive enzymatic activity.^17^^,^^18^ However, a statistically significant difference in the ratio between the two groups was not found.

Entropy values of the infiltrate were analyzed to determine if *Pseudomonas* leads to a different entropy level within the lesion. Analysis of OCT images with the greatest differences was performed. We hypothesized that lesions in *Pseudomonas* keratitis would lead to higher entropy, as the immune reaction is higher and the infiltrates tend to be more dense. However, this was not confirmed in this study.

**Figure 7.**
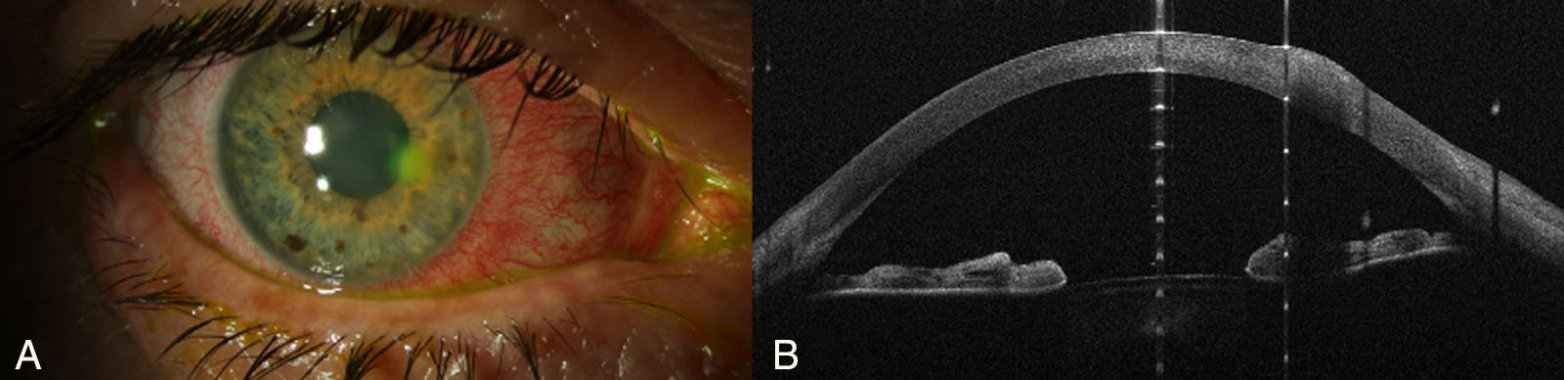
Comparison of diagnostic imaging in a patient with contact lens–associated keratitis and symptom onset within the last 24 hours. Both masked reviewers accurately identified this case as *Pseudomonas* keratitis. (A) Slit-lamp photograph revealing a paracentral corneal infiltrate and conjunctival hyperemia; the anterior chamber is not affected. (B) AS-OCT image of the right eye demonstrating an increase in corneal thickness (tissue gain) at the lesion area.

**Figure 8.**
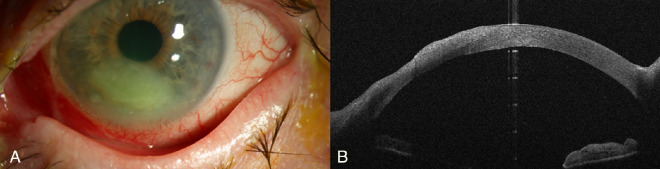
Comparison of diagnostic imaging in a patient with foreign body–associated *Staphylococcus epidermidis* keratitis. Symptom onset occurred within 48 hours. Both masked reviewers misidentified this case as *Pseudomonas* keratitis. (A) Slit-lamp photograph showing an inferior ulcerative infiltrate in Gram-positive keratitis. (B) Corresponding AS-OCT showing loss of corneal tissue in the infiltrate area.

*Pseudomonas* infections often progress rapidly and display destructive enzymatic activity leading to characteristic necrotic features. On the other hand, Gram-positive keratitis typically presents as well-defined, cream-colored or gray–white stromal infiltrates. Although our study focused on utilizing AS-OCT to distinguish between these bacterial etiologies, additional diagnostic methods are also important. External eye photography has been proposed as a complementary tool to validate AS-OCT findings and enhance diagnostic accuracy.^19^ One experienced masked reviewer (PL) demonstrated commendable accuracy in diagnosing *Pseudomonas* keratitis based on imaging (slit-lamp photography and AS-OCT), with a notable four out of seven cases being correctly identified. In contrast, the second reviewer (KW), who assessed OCT and slit-lamp images separately, displayed a slightly lower accuracy in identifying *Pseudomonas* keratitis (three correctly identified based on OCT and four correctly identified based on slit-lamp photography). Additionally, there were instances where Gram-positive keratitis cases were misinterpreted as *Pseudomonas* keratitis (five incorrectly identified based on OCT and four incorrectly identified based on slit-lamp photography). The initial reviewer (PL), who was assessing both modalities, might have benefited from a more comprehensive evaluation, taking into account information from both imaging modalities. However, the second reviewer (KW), who separately examined the photographs, might have encountered problems resulting in a marginally lower level of diagnostic accuracy (see Figs. 7, 8). However, the misinterpretation of some Gram-positive cases as *Pseudomonas* emphasizes the challenges associated with the use of only AS-OCT or slit-lamp photography for diagnosis.

One of the strengths of this study is that patients were not receiving therapy at the time of enrollment, so only therapy-naïve eyes were included. A limitation of this study was the relatively small sample size. Multicenter studies with *Pseudomonas* subtype analysis may contribute to a better understanding of pathognomonic patterns in OCT. One weakness was that the tissue loss/gain ratio was calculated with measurements from the same eye. The proportions are skewed because the cornea is thicker temporally than nasally. Another limitation is that this study did not focus on different groups of *Pseudomonas* bacteria depending on their exotoxins.^20^ However, this differentiation is relevant, as all exotoxins require individualized therapies, especially regarding steroids.

Our study not only establishes the utility of AS-OCT in differentiating these bacterial etiologies based on quantitative measurements of infiltrate dimensions, corneal thickness, and tissue loss/gain but also underscores its potential as a valuable prognostic tool. By visualizing the structural changes within the cornea, AS-OCT can offer insights into the severity of tissue involvement and progression, which can guide prognostication and management decisions. In the future, a larger sample will be included and differences among several exotoxins will be evaluated.

## Figures and Tables

**Figure A1.**
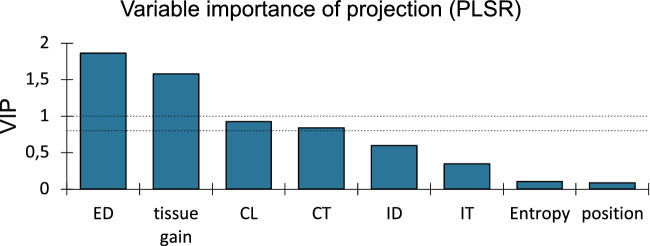
Partial Least Squares Regression (PLSR) Analysis Incorporating Multiple Parameters: Epithelial Defect, Tissue Gain, Contact Lens Usage, Corneal Thickness, Infiltrate Diameter, Infiltrate Thickness, Entropy, and position of the lesion (=distance to center).

**Figure A2.**
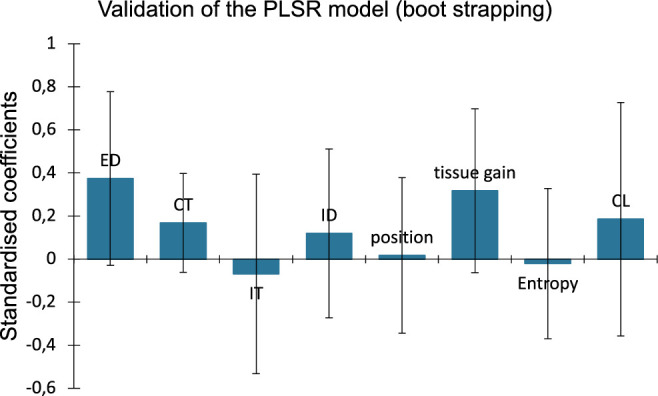
PLSR Model Validation with Bootstrapping: Assessing Model Stability and Predictive Performance through Bootstrap Resampling

## Appendix. Appendix

 ^9^,^10^
